# Are Cochlear Implants Indicated in the Advanced Aging Population?

**DOI:** 10.1002/lary.70521

**Published:** 2026-03-31

**Authors:** India Jackson, Tiffany Peng Hwa, Michael Ruckenstein

**Affiliations:** ^1^ Perelman School of Medicine University of Pennsylvania Philadelphia Pennsylvania USA; ^2^ Department of Otorhinolaryngology–Head and Neck Surgery University of Pennsylvania Philadelphia Pennsylvania USA

**Keywords:** cochlear implants, elderly, otology/neurotology, sensorineural hearing loss

## 1 Background

Older adults with severe to profound sensorineural hearing loss face substantial functional limitations, including social withdrawal and cognitive decline. As patients are more commonly living into their 90s, evidence‐based recommendations are required to inform providers as to if their patients would benefit from cochlear implantation. These recommendations would help resolve bias that might be associated with performing an elective surgery on octo‐ and nonagenarians.

## 2 Literature Review

Across studies, “elderly” includes adults over 60, with the strongest evidence derived from larger, more recent cohort studies of octogenarians and nonagenarians. Multiple large cohort studies show that adults aged 80 years and older achieve meaningful speech perception scores similar to the outcomes of younger cohorts [1]. Daily device use remains consistent, exceeding 8 h a day, and serious postoperative complications are uncommon, with major complication rates under 5% and transient dizziness under 10% in retrospective and observational cohorts spanning approximately 2010–2024 (Table 1) [1, 2, 3].

**TABLE 1:** Summary of included studies.

Study	Design	Population (total/elderly)	Elderly age cutoff	Outcomes	Key findings	Notes/significance
Zhan et al. (2025)	Retrospective cohort	221 total (80–89: *n* = 171; ≥ 90: *n* = 50)	≥ 80 years	Speech perception (CNC, AzBio) Cognition (not specified) QOL Device use Tinnitus and balance problems	Speech perception: 1 year speech scores were similar in 80–89 vs. ≥ 90 → no meaningful difference by age subgroup Cognition: Not assessed Complications/other: Persistent balance symptoms decreased by 1 year; average daily device use was high (> 11 h)	Largest ≥ 80 cohort to date. Shows robust speech improvement even ≥ 90 years, stable device use, and low complication risk Cognition and comorbidity did not affect benefit.
Ismail et al. (2025)	Systematic review	22 studies; included cohorts ranging from 24 to 925 participants with BL severe to profound SNHL	≥ 60 years	Speech perception (not specified) Cognition (not specified) QOL Safety	Speech perception: Improved substantially across studies; QOL improved Cognition: Cognitive performance improved modestly, with gains in executive function and memory Complications/other: Major complications were uncommon; transient dizziness was uncommon; daily CI use remained high (> 10 h)	Comprehensive systematic review (2010–2024) summarizing 22 modern studies across multiple domains. Confirms strong safety and benefit profile for CIs in adults ≥ 60 years. Highlights need for equitable access and standardized geriatric referral pathways.
Kim et al. (2025)	Retrospective cross‐sectional study	104 total (52 CI users ≥ 70; 52 age/sex matched healthy controls)	≥ 70 years	Speech intelligibility (monosyllabic [MSW], disyllabic [DSW], sentence percept. tests [SPT], voxel‐based morphometry) Cognition (not specified)	Speech perception: Reduced gray matter in cerebellar Crus I was associated with poorer postop speech scores Cognition: Not assessed Complications/other: Age at implantation was not associated with CI results	Important neuroimaging study identify cerebellar (Crus I) atrophy as an independent predictor of poor CI performance in elderly adults. Suggest preop MRI eval of cerebellar vL may improve prognostic counseling.
Hallin et al. (2023)	Cross‐sectional observational study (of existing CI users)	43 total CI users aged 85–97 (mean age ~88); mean age at implantation ~79 years	≥ 85 years	Speech perception (not specified) Cognition (not specified) Self‐reported benefit Handling difficulty Complications	Speech perception: Speech understanding was generally maintained Cognition: Not assessed Complications/other: Most patients reported meaningful benefit and full‐time use; new dizziness was rare; some patients reported device management difficulties	Shows that even patients ≥ 85 years are using their CIs daily, perceive clear benefit, and are largely safe to implant. The elderly population tend to need more support with device management.
An et al. (2023)	Systematic review + meta‐analysis	20 studies; 648 CI subjects with post‐lingual SPHL	≥ 60 years (mean ≥ 55 years in all studies)	Speech perception (not specified) Cognition (global, executive, verbal, memory) Pre‐ and post‐implant at 6 and 12 months	Speech perception: Improved at 6 and 12 months Cognition: Executive function improved through 12 months; verbal memory improved by 6 months and was sustained; global function improved at 12 months Complications/other: Not reported	Demonstrates long‐term cognitive and speech benefit after CI in older adults. Suggests reduced “listening effort” and neuroplastic reallocation underlie improvement which supports early CI for dementia risk reduction.

In addition to auditory outcomes, contemporary evidence associates cochlear implantation with modest improvements in cognitive domains such as memory and executive function. Meta‐analyses show stabilization or improvement in cognition after implantation, with the greatest gains often observed in individuals with prior baseline deficits [4]. Systematic reviews of older adult CI recipients reinforce these findings. Speech perception improvements, enhanced quality of life, and broad safety data converge across studies of adults aged 60 and above. These reviews support age‐neutral referral practices and emphasize patient‐specific factors such as overall health, cognitive status, and personal goals.

Radiographic and prognostic studies suggest that poorer outcomes relate more to neuroanatomic or cognitive variables rather than chronological age. Emerging evidence seeks to identify neuroimaging biomarkers that stratify outcomes beyond age alone, including findings such as cerebellar Crus I atrophy, which has been associated with poor speech perception after implantation [5]. Identifying such markers can guide counseling and expectation management without excluding older adults from candidacy pathways.

## 3 Best Practice Summary

Cochlear implantation is indicated for older adults with severe to profound hearing loss when they meet standard candidacy criteria. The results of elderly cohorts mirror that of younger patient cohorts and reinforce that age alone should not disqualify a patient from referral or implantation [1]. Current evidence supports meaningful benefit, safety, and quality of life improvement with cochlear implantation across older adults, from those in their 60s through octogenarians and nonagenarians. Providers should assess medical fitness, cognitive status, and functional goals to guide individualized decision making. Continuous support, rehabilitation, and age‐appropriate assistance with device management remain key to optimizing outcomes, particularly in patients of advanced age [2, 3]. Reports of outcomes in both octogenarians and nonagenarians confirm that the risk from advanced age alone does not outweigh the benefit for appropriate candidates [1, 2, 3, 4, 5].

## 4 Level of Evidence

Based on systematic reviews, cohort studies, and observational studies, the level of evidence is N/A.

## Funding

The authors have nothing to report.

## Conflicts of Interest

The authors declare no conflicts of interest.

## Data Availability

No new data were generated or analyzed in this study. All data are derived from previously published sources cited in the manuscript.
